# Heart Failure Biomarkers—Pathophysiology, Diagnosis, Prognosis and Clinical Relevance

**DOI:** 10.3390/ijms26199740

**Published:** 2025-10-07

**Authors:** Bianca-Ștefania Profire, Florentina Geanina Lupașcu, Cristian Stătescu, Victorița Șorodoc, Radu-Andy Sascău, Lenuța Profire, Laurențiu Șorodoc

**Affiliations:** 1 Faculty of Medicine, Grigore T. Popa University of Medicine and Pharmacy Iași, 16 University Street, 700115 Iași, Romania; bianca-stefania.profire@umfiasi.ro (B.-Ș.P.); cristian.statescu@umfiasi.ro (C.S.); victorita.sorodoc@umfiasi.ro (V.Ș.); laurentiu.sorodoc@umfiasi.ro (L.Ș.); 2 Faculty of Pharmacy, Grigore T. Popa University of Medicine and Pharmacy Iași, 16 University Street, 700115 Iași, Romania; florentina-geanina.lupascu@umfiasi.ro (F.G.L.); lenuta.profire@umfiasi.ro (L.P.); 3 Institute for Cardiovascular Diseases “Prof. Dr. George I.M. Georgescu”, 50 Carol I Boulevard, 700503 Iași, Romania; 4 “Sf. Spiridon” Clinical Emergency Hospital, 1 Independence Boulevard, 700111 Iași, Romania

**Keywords:** heart failure, pathophysiology, biomarkers, diagnosis, prognosis

## Abstract

Heart failure (HF) is a complex clinical syndrome characterized by impaired cardiac function and maladaptive neurohormonal activation, representing one of the leading causes of morbidity, hospitalization, and mortality worldwide. Both its incidence and prevalence continue to rise, largely as a consequence of population aging and the increasing burden of cardiovascular risk factors. The pathogenesis of HF is multifactorial, involving a dynamic interplay between inflammation and neurohormonal activation, ultimately leading to cardiac remodeling, diastolic dysfunction, and impaired cardiac output. In this context, numerous biomarkers have been investigated for diagnosis and prognosis utility in patients with HF. According to their underlying pathophysiological mechanisms, biomarkers in HF can be broadly categorized as indicators of inflammation, oxidative stress, cardiac remodeling, myocardial stress, neurohormonal activation, and cardiomyocyte injury. This review summarizes current knowledge on the pathophysiological basis of HF and highlights the diagnostic and prognostic relevance of circulating biomarkers, emphasizing their role in linking disease mechanisms with clinical management.

## 1. Introduction

Heart failure (HF) is a clinical syndrome characterized by cardinal symptoms (dyspnea, fatigue), which may be accompanied by specific signs (peripheral edema, pulmonary rales, elevated jugular venous pressure). It is caused by a structural or functional cardiac abnormality that impairs the ability to maintain an adequate cardiac output at rest or during exertion, or can only ensure sufficient tissue perfusion at the cost of elevated left ventricular filling pressures [1].

Identifying the etiological substrate that led to the development of cardiac dysfunction is essential after establishing the clinical diagnosis of HF, since initiating a targeted treatment can improve both prognosis and disease progression.

HF is the leading cause of death worldwide, with an incidence of approximately 10 cases per 1000 individuals aged over 65 years [1,2]. Estimated survival rates after diagnosis for HF patients are 87%, 73%, 57%, and only 35% at 1, 2, 5, and 10 years, respectively [3]. Globally, this clinical syndrome affects approximately 65 million people, with alarming projections for the coming years [4]. In the United States, it is estimated that by 2030, 8 million individuals will be diagnosed with HF. The high incidence and prevalence of HF can be partially explained by the improved survival of patients with ischemic heart disease due to advances in both pharmacological and interventional therapies, as well as by the increasing awareness and management options for HF. Another important aspect is the long-term prognosis of children with congenital heart defects; although therapeutic advances have enabled more than 90% to reach adulthood, there remains a substantial lifetime risk of developing cardiac dysfunction [5].

In Europe, cardiovascular diseases are the leading cause of mortality, accounting for 55% of deaths in women and 43% in men, when analyzed separately by sex. The remaining causes of death in each group are mainly due to cancer, respiratory diseases, and other non-cardiovascular conditions. Numerically, these conditions cause over 4 million deaths annually across the continent [4]. Furthermore, the number of new cases recorded each year is estimated at 6 million in the European Union and over 11 million in the whole continent, with an incidence ranging from 2.5 cases per 1000 individuals (Italy, Denmark) to 6.5 cases per 1000 individuals (Germany, Estonia, Lithuania). The prevalence of HF also varies widely, from under 12 cases per 1000 inhabitants in Spain and Greece to over 30 cases per 1000 inhabitants in Lithuania and Germany [6]. According to the Global Burden of Disease Study 2021, cardiovascular diseases, including HF, remain among the leading global causes of disability-adjusted life years (DALYs). Between 1990 and 2021, the absolute number of DALYs attributable to cardiovascular diseases increased substantially, mainly due to population growth and ageing, while age-standardized DALY rates declined, reflecting progress in prevention and management [7]. More specifically, data from the GBD 2021 analysis dedicated to HF showed that the disability burden measured as years lived with disability (YLDs) more than doubled from 2.4 million in 1990 to 5.3 million in 2021, whereas the age-standardized YLD rate increased only slightly (from 61 to 65 per 100,000 population) [8].

In addition, the prevalence is expected to rise due to population ageing and improved survival after other cardiovascular diseases. This trend is further accentuated by the increasing burden of risk factors, including metabolic disorders (obesity, diabetes, hypertension), genetic predisposition, chronic pulmonary and inflammatory diseases, and chronic infections, as well as toxic exposures such as alcohol abuse, cocaine, and cardiotoxic therapies (e.g., anthracyclines or trastuzumab in breast cancer treatment) [9,10].

According to left ventricular ejection fraction (LVEF), HF is classified into three major categories: HF with preserved ejection fraction (HFpEF; LVEF ≥ 50%), HF with mildly reduced ejection fraction (HFmrEF; LVEF 41–49%), and HF with reduced ejection fraction (HFrEF; LVEF ≤ 40%) [11]. Based on the observation that 10% to 60% of patients with HFrEF experience an improvement in LVEF, a new category, HF with improved EF (HFimEF), has been recently introduced. HFimEF is defined by an absolute increase of LVEF of ≥10% to a value higher than 40%. Compared with HFrEF, HFimpEF is associated with a more favorable prognosis; however, accumulating evidence indicates a continued risk of relapse and adverse clinical events in some patients [12].

Among all HF phenotypes, HFpEF remains particularly challenging, being associated with a considerable burden of morbidity and mortality, with hospitalization rates of up to 80% and mortality rates approaching 50% within five years [11].

The etiology of HF is multifactorial, and a precise identification of the underlying cause of myocardial dysfunction is essential for accurate diagnosis, risk stratification, and optimal patient management [13]. It is well established that two-thirds of HF cases are attributable to chronic conditions, including ischemic heart disease, arterial hypertension, valvular heart disease, and cardiomyopathies, among others [14,15]. Moreover, HFpEF is closely associated with metabolic syndrome, whereas HFrEF is more frequently linked to ischemic heart disease and myocardial injury [16,17]. In the HFpEF phenotype, the presence of non-cardiovascular comorbidities such as anemia, diabetes mellitus, chronic kidney disease, chronic lung disease, sleep apnea, and cognitive or neurological disorders negatively affects prognosis, independently of HF status and progression [18,19].

The etiology of HF varies according to geographic region. In high-income countries, ischemic heart disease and arterial hypertension remain the leading causes of HF, although the number of hypertensive heart disease cases has begun to decline as a result of effective primary prevention strategies for hypertension [20]. Conversely, an increase in HF cases secondary to valvular heart disease is anticipated, in the context of population aging. Rheumatic heart disease continues to represent a major public health concern in some low-income countries in Africa and Asia, while Chagas disease remains an important cause of HF in South America [21].

Referring to HF management, first-line therapy in HF, regardless of the presence or absence of diabetes mellitus or other comorbidities, includes beta-blockers, angiotensin-converting enzyme inhibitors (ACEIs) or angiotensin II receptor blockers, and mineralocorticoid receptor antagonists. More recently, the therapy has been expanded with other additional drug classes, angiotensin receptor-neprilysin inhibitors (ARNIs, e.g., sacubitril/valsartan), sodium–glucose co-transporter 2 (SGLT2) inhibitors (dapagliflozin, empagliflozin) and soluble guanylate cyclase (sGC) stimulators (vericiguat) [22,23]. Current evidence indicates that SGLT2 inhibitors, sacubitril/valsartan and vericiguat provide clinical benefits in HF, with variations across specific patient subgroups. Dapagliflozin and empagliflozin demonstrated consistent reductions in cardiovascular death and HF hospitalizations, particularly in elderly patients, those with chronic kidney disease (CKD), diabetes mellitus, or ischemic HFrEF. Sacubitril/valsartan and vericiguat were effective in CKD and advanced NYHA functional classes. Overall, these therapies complement each other, offering tailored options depending on comorbidities and clinical profile [24].

## 2. Pathophysiology of Heart Failure

**Figure 1:**
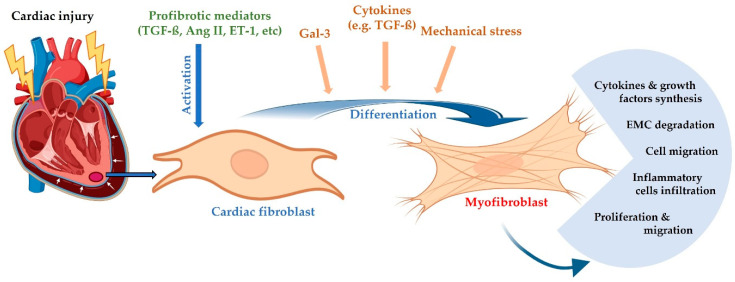
Differentiation of cardiac fibroblasts into myofibroblasts and their response to myocardial injury.

The pathogenesis of HF is complex, involving the interconnection between inflammation and neurohormonal activation, ultimately leading to cardiac remodeling, diastolic dysfunction, and impaired cardiac output [25]. In the context of pathological conditions associated with HF, factors such as systemic inflammation, hypoxia, cardiomyocyte dysfunction and death, mechanical stress, and the release of profibrotic cytokines promote the migration and proliferation of cardiac fibroblasts in affected areas, as well as their differentiation into active myofibroblasts [5,26]. These myofibroblasts acquire a pronounced contractile phenotype and stimulate the secretion of profibrotic signaling factors, including transforming growth factor beta (TGF-β), tumor necrosis factor alpha (TNF-α), and angiotensin II, which in turn can induce significant alterations in myocardial architecture and promote cardiomyocyte hypertrophy (Figure 1) [1].

Myofibroblasts are the most important cell type responsible for the structural characteristics of the extracellular matrix, contributing to the development of interstitial and perivascular fibrosis, which stiffens the myocardium and also leads to collagen-rich scar formation at sites of acute injury. This type of fibrosis can further exacerbate myocardial stiffening and preexisting diastolic dysfunction, previously triggered by systemic inflammation or alterations in cellular energy metabolism. Taken together, these changes promote an adaptive hypertrophic response of the left ventricle, which compromises its relaxation capacity, ultimately leading to diastolic dysfunction [1,27].

### 2.1. Inflammation

Compared with healthy individuals, patients with HF exhibit elevated circulating levels of inflammatory markers such as TNF-α, interleukin-6 (IL-6), E-selectin, and intercellular adhesion molecule 1 (ICAM-1), along with evidence of inflammatory cell infiltration of cardiomyocytes at the endomyocardial level. Physiological aging is accompanied by a chronic, low-grade inflammatory process termed *inflammaging*, with elderly individuals displaying higher serum concentrations of TNF-α, IL-6, and interleukin-1 beta (IL-1β) compared with younger individuals [28]. This chronic inflammatory state is sustained by cytokine-producing senescent cells, pro-inflammatory immune remodeling, and alterations in the gut microbiota. Moreover, inflammatory activation is more pronounced in women, reflected by an exaggerated immune response, an increased susceptibility to autoimmune diseases, and myocardial overexpression of immune-related genes. Pregnancy and pregnancy-related conditions, such as gestational diabetes and preeclampsia, also contribute to inflammatory activation, adding to the age-related increase in cardiometabolic risk. The association between HF, inflammation, and cardiometabolic risk factors, such as obesity, diabetes mellitus, and hypertension, is well supported by scientific evidence [4].

Inflammation in HF is closely linked to oxidative stress, both processes being associated with common comorbidities, including advanced age, obesity, and diabetes mellitus. TNF-α and IL-6 stimulate nicotinamide adenine dinucleotide phosphate oxidase (NADPH oxidase, NOX) activity in endothelial cells, inflammatory cells, and cardiomyocytes, leading to the production of reactive oxygen species (ROS) [10,28]. Experimental studies in animal models of HF and hypertension have shown that inhibition of NOX markedly improves diastolic dysfunction and reduces ROS overproduction. In turn, oxidative stress further amplifies the production of inflammatory mediators.

Macrophages, particularly the M1 subtype, play a pivotal role in the inflammatory process in HF. These cells are activated by adipocyte-derived factors, hyperglycemia, and aging. Interestingly, elevated concentrations of M2 macrophages have also been observed in HF; however, it remains unclear whether disease severity depends on the relative proportions of these subtypes. In addition, CD4+ T lymphocytes have an important role in modulating the pro-inflammatory response in HF, with reduced activity of anti-inflammatory regulatory T cells (Tregs) being associated with an increased proportion of pro-inflammatory Th1 cells [4].

Another inflammatory pathway implicated in HF pathophysiology involves mitogen-activated protein kinase (MAPK) and its downstream signaling targets, including c-Jun N-terminal kinases (JNKs), extracellular signal-regulated kinases (ERKs), and p38 mitogen-activated protein kinase (p38-MAPK). Inflammation and oxidative stress induced by TNF-α and ROS stimulate JNK and p38-MAPK activity in cardiomyocytes and fibroblasts. In turn, p38-MAPK activation exacerbates inflammation by increasing IL-6 and TNF-α levels [29].

### 2.2. Neurohormonal Activation

The renin–angiotensin–aldosterone system (RAAS) plays a central role in HF, becoming activated secondary to renal hypoperfusion and sympathetic nervous system stimulation. Renin, synthesized by juxtaglomerular cells in the kidney in response to hypotension, hypovolemia, or sympathetic stimulation, converts angiotensinogen (hepatically synthesized) into angiotensin I (Ang I), which is subsequently converted to angiotensin II (Ang II) by angiotensin-converting enzyme (ACE), secreted primarily by the lungs [21,25].

In the cardiovascular system, Ang II exerts multiple initially adaptive effects that later become maladaptive, driving HF progression and symptom worsening. Ang II stimulates cardiomyocyte hypertrophy, promotes the development of interstitial fibrosis, and, in advanced stages, induces apoptosis, leading to deterioration of myocardial structure and function and subsequent organ dysfunction [21].

The sympathetic branch of the autonomic nervous system exerts its cardiovascular effects through β1-, β2-, and α1-adrenergic receptors, closely linked to RAAS activation. Prolonged stimulation of adrenergic receptors results in myocardial cardiotoxicity, progression to systolic dysfunction, and increased arrhythmogenic risk. Peripherally, β1- and α1-receptor activation triggers secondary RAAS activation, promoting vasoconstriction and sodium–water retention. In the early stages of HF, catecholamines exert compensatory effects by increasing heart rate and contractility (positive chronotropic and inotropic effects). However, long-term increases in cardiac output come at the cost of elevated myocardial oxygen consumption, predisposing to ischemia, ventricular remodeling, and higher arrhythmia risk [10,30].

### 2.3. Cardiac Remodeling

Cardiac remodeling involves a series of structural and functional changes within the myocardium, primarily characterized by cardiomyocyte hypertrophy and myocardial fibrosis [31].

Initially, cardiac hypertrophy in response to pathological stress serves an adaptive role. However, under conditions of sustained injury, this response becomes maladaptive, leading to progressive wall thickening, dilation, and remodeling extending to adjacent vascular structures. In advanced stages, these changes are accompanied by cell loss through apoptosis or necrosis, contributing to the development of cardiac dysfunction [32].

Cardiac hypertrophy is associated with increased protein synthesis, disorganization of sarcomeric architecture, and activation of genes encoding atrial natriuretic peptide (ANP), B-type natriuretic peptide (BNP), β-myosin heavy chain (β-MHC), and skeletal muscle α-actin. Inflammatory processes within the myocardium modulate key pathways involved in hypertrophy, leading to increased wall stiffness and diastolic dysfunction. Persistent hypertrophy is frequently associated with HF, arrhythmias, and, in advanced cases, sudden cardiac death [33].

The hypertrophy characteristic of HF is concentric, defined by myocardial wall thickening and transverse cardiomyocyte enlargement. This remodeling pattern develops in response to pressure overload, reducing ventricular compliance and impairing diastolic function. At the cellular level, TNF-α activates calcium-dependent pro-hypertrophic transcription factors such as nuclear factor of activated T cells (NFAT). IL-6 and IL-1 also promote cardiac hypertrophy via the Janus kinase/signal transducer and activator of transcription (JAK/STAT) pathway [4].

A crucial role is played by G protein-coupled receptors (GPCRs), widely expressed throughout the body, mediating cellular responses to various activated ligands, including vasoconstriction, vasodilation, angiogenesis, and increased myocardial contractility. In HF, GPCR expression is reduced, with loss of these receptors being linked to increased mortality, enhanced inflammatory responses, and expansion of infarct size in ischemic heart disease [34].

Fibrosis is defined as excessive deposition of extracellular matrix (ECM) proteins, particularly collagen type I and fibronectin, within tissue parenchyma, impairing organ function. The cardiac ECM is a complex three-dimensional structure composed of collagen type I (~80%) and type III (~10%), with smaller amounts of types V and VI collagen, laminin, elastin, glycosaminoglycans, and proteoglycans. The ratio between collagen types I and III varies across myocardial regions and under physiological versus pathological conditions [29].

Two forms of fibrosis are distinguished: (i) reparative fibrosis, replacing dead cardiomyocytes with collagen-based scar tissue, and (ii) reactive fibrosis, involving gradual ECM protein deposition without direct cell death, typically around blood vessels, as a maladaptive response to hemodynamic, inflammatory, or neurohormonal stimuli [35].

Myocardial infarction (MI) is a classic example of a condition that triggers reparative fibrosis, and it is important to note that the resulting scar tissue lacks contractile properties. Although reparative fibrosis plays a crucial role in preserving structural integrity after myocardial infarction, over time it contributes to expansion of the interstitial compartment through excessive accumulation of ECM proteins, with significant functional consequences. In this context, the healing process can be divided into two distinct phases: an inflammatory phase, responsible for the removal of necrotic tissue, and a proliferative phase, in which myofibroblasts produce ECM proteins as a compensatory response to cell death [28,35].

In contrast, reactive fibrosis occurs predominantly in non-ischemic settings, most often involving inflammation and oxidative stress. Common pathological conditions such as hypertension, diabetes mellitus, and obesity may, over time, promote the progressive accumulation of ECM proteins in both the interstitial and perivascular compartments, ultimately leading to myocardial stiffness and dysfunction. Endomyocardial biopsy studies have shown that 95% of HFpEF patients present with diffuse interstitial and perivascular fibrosis [28,35].

The molecular mechanisms underlying fibrosis involve TNF-α, IL-6, and the NOD-like receptor family pyrin domain-containing 3 (NLRP3) inflammasome, which mediates smooth muscle actin expression, thereby driving the differentiation of fibroblasts into contractile myofibroblast phenotypes [26,28]. Subsequently, at the interstitial level, myofibroblasts secrete procollagen, whose accumulation leads to excessive ECM deposition and fibrosis development. In addition, TNF-α promotes collagen cross-linking via activation of the lysyl oxidase (LOX) enzymatic pathway, a mechanism that further increases myocardial stiffness [4]. Fibrosis contributes to the onset and progression of HF through multiple maladaptive mechanisms: it increases myocardial stiffness, induces disorganized and dyssynchronous contractions, disrupts electrical impulse conduction, and exacerbates tissue hypoxia [27,31].

### 2.4. Oxidative Stress

Mitochondrial oxidative stress, which arises from an imbalance between ROS levels and the activity of endogenous antioxidant systems, leads to contractile dysfunction, thereby contributing to the onset and progression of HF. Oxidative stress is also among the key factors involved in the transition from adaptive hypertrophy to the reversal of compensatory mechanisms and the development of HF. Ischemic heart disease impairs mitochondrial function, reducing the activity of superoxide dismutase (SOD) and glutathione peroxidase (GPx), which results in elevated hydrogen peroxide (H_2_O_2_) concentrations [1]. Furthermore, cellular injury within the ischemic zone promotes neutrophil infiltration, which intensifies oxidative stress through the production of ROS, particularly superoxide anion, as well as the release of pro-inflammatory cytokines (TNF-α) and the activation of NADPH oxidases (NOX2, NOX4). In HF patients, elevated levels of oxidative stress biomarkers (e.g., 8-iso-prostaglandin F_2_α) and secondary lipid peroxidation products have been identified, with these levels correlating with the severity of ventricular dysfunction [10].

Oxidative stress may also be mediated by the sympathetic nervous system, RAAS, and endothelin-1. In animal models, chronic norepinephrine administration in rats has been shown to induce oxidative stress, while isoproterenol (ISO) enhances lipid peroxidation and reduces myocardial GPx concentrations [32]. In addition, Ang II, through binding to its specific receptors, activates plasma membrane NADPH oxidase, thereby increasing superoxide anion concentrations. Although this phenomenon was initially observed in vascular smooth muscle cells, evidence also supports elevated NADPH oxidase expression in cardiomyocytes. Ang II further activates mitochondrial ATP-sensitive potassium channels, enhancing ROS synthesis and consequently exacerbating oxidative stress [1].

Endothelin-1 is a 21-amino acid vasoconstrictor peptide with a key role in vascular injury. It has affinity for two receptor subtypes with distinct localization: the ETA receptor, expressed predominantly in vascular smooth muscle cells, and the ETB receptor, found mainly in endothelial cells. Under physiological conditions, endothelin-1 is synthesized by endothelial cells [25]. In pathological states, stimuli such as ischemia, elevated catecholamines, and Ang II promote endothelin-1 synthesis in both endothelial cells and cardiomyocytes. In cardiomyocytes, which predominantly express ETA receptors, endothelin-1 induces oxidative stress, as well as tissue remodeling and fibrosis, through fibroblast activation and proliferation [25,36].

Another factor involved in oxidative stress generation is selenium, which acts as a cofactor for antioxidant enzymes, including GPx and thioredoxin reductase (TrxR). Severe selenium deficiency impairs endogenous antioxidant defenses, leading to an endemic cardiomyopathy known as Keshan disease [37].

Drug-induced cardiotoxicity, particularly from doxorubicin, represents another important oxidative stress-mediated mechanism of HF. Histological analyses of endomyocardial biopsies from patients with doxorubicin-induced cardiac dysfunction have revealed multiple cellular structural alterations, including reduced myofibril content, sarcoplasmic hypertrophy, cytoplasmic vacuolization, mitochondrial swelling, and an increased number of lysosomes [1]. Experimental studies on cardiac tissue extracts have demonstrated that doxorubicin induces mitochondrial generation of H_2_O_2_ and superoxide anions. These ROS, in turn, trigger cardiomyocyte apoptosis, effects that can be mitigated by antioxidant agents [5].

### 2.5. Genetic and Environmental Factors

HF pathogenesis is also modulated by genetic predisposition and environmental influences, which interplay to affect the development and progression of the disease. Numerous gene variants and mutations have been linked to HF, both indirectly by increasing the prevalence of risk factors and directly by causing primary cardiomyopathies. Large-scale sequencing studies have shown that pathogenic or likely pathogenic variants are present in up to 15–20% of patients with dilated cardiomyopathy (DCM), underscoring the contribution of inherited forms to the HF spectrum. Among these, truncating mutations in the TTN gene, encoding titin, represent the most frequent cause of genetic DCM, accounting for 20–25% of familial cases and significantly pre-disposing carriers to left ventricular dilation and impaired systolic function. Variants in MYH7 (β-myosin heavy chain) and MYBPC3 (myosin-binding protein C) are strongly associated with hypertrophic cardiomyopathy. Moreover, mutations in LMNA, encoding nuclear envelope protein lamin A/C, are linked not only to DCM but also to conduction abnormalities and malignant ventricular arrhythmias, often leading to early-onset HF. Importantly, the penetrance and severity of these mutations are modulated by age, sex, and environmental exposures, suggesting a dynamic gene-environment interaction [38].

Beyond rare monogenic forms, polygenic susceptibility also contributes substantially to HF risk. Genome-wide association studies (GWASs) have identified over 40 loci associated with HF, implicating pathways related to cardiomyocyte contractility, extracellular matrix turnover, and systemic risk factors such as hypertension and diabetes. Polygenic risk scores derived from these loci have been shown to improve prediction of HF incidence, particularly when integrated with traditional clinical risk factors [39].

Environmental factors also play a pivotal role in HF pathophysiology. Chronic exposure to air pollution, particularly fine particulate matter (PM_2.5_), has been linked to increased HF incidence, hospitalizations, and mortality. A meta-analysis of 35 studies found that each 10 μg/m^3^ rise in PM_2.5_ (or PM_10_) concentration is associated with approximately a 2% increase in HF-related hospitalizations and deaths. Persistent exposure to high levels of environmental noise (e.g., road traffic noise) is another emerging risk factor; epidemiologic data indicate about a 4% higher risk of developing HF per 10 dB increase in chronic noise exposure. These environmental stressors likely contribute to HF via mechanisms of oxidative stress, inflammation, and endothelial dysfunction, which exacerbate myocardial remodeling and impair cardiac function [40]. Furthermore, unhealthy lifestyle factors (such as smoking, poor diet, and physical inactivity) often interact with genetic susceptibility and environmental exposures to accelerate comorbid conditions (e.g., atherosclerosis, diabetes, hypertension) that predispose individuals to HF [41].

## 3. Diagnostic and Prognostic Biomarkers

For a long time, the symptomatology of HF was attributed primarily to central hemodynamic alterations, with reduced cardiac performance considered the main cause of dyspnea—through increased pulmonary capillary pressure—as well as physical fatigue, secondary to decreased cardiac output and peripheral hypoperfusion. Subsequently, it became evident that the hemodynamic hypothesis does not fully account for the clinical presentation of HF, leading to the formulation of new explanatory models [42].

Biomarkers are highly valuable biomolecules for assessing physiological processes, pathological states, and responses to pharmacological therapy. To be clinically useful and effective, a biomarker must fulfill several criteria, of which the most important are: (i) high sensitivity and specificity, and (ii) the quantification method must be rapid, accurate, reproducible, standardized, and cost-effective [42,43].

Several biomarkers have emerged as robust predictors of HF incidence, severity, and outcomes, including NT-proBNP, troponin, soluble ST2, and galectin-3, among others. Their incremental prognostic value has been demonstrated across diverse clinical cohorts. From a methodological perspective, currently available assays for HF biomarkers offer very low limits of detection (LoD), generally in the pg/mL to ng/mL range. For instance, the analytical LoD is approximately 10 pg/mL for NT-proBNP and 2.8 ng/mL for soluble ST2, values that are far below the typical concentrations encountered in clinical HF populations. This ensures that these biomarkers can be measured reliably within clinically relevant ranges, supporting their use for both diagnostic and prognostic assessment [44].

Current guidelines recommend natriuretic peptides (BNP or its precursor NT-proBNP) as reference biomarkers for HF. However, their levels may be influenced by multiple clinical factors, including age, sex, renal function, body mass index (particularly obesity), thyroid status, pulmonary disease, and anemia. In this context, complementary evaluation of other biomarkers is warranted, particularly those reflecting different pathophysiological mechanisms involved in HF [43,45].

For a novel biomarker to be considered useful in the management of HF patients, it must demonstrate clinical value in one or more of the following domains: diagnosis, prognostic estimation through risk stratification, monitoring of therapeutic response, or early identification (within 30 days) of major adverse events such as death or acute myocardial infarction [45].

**Figure 2:**
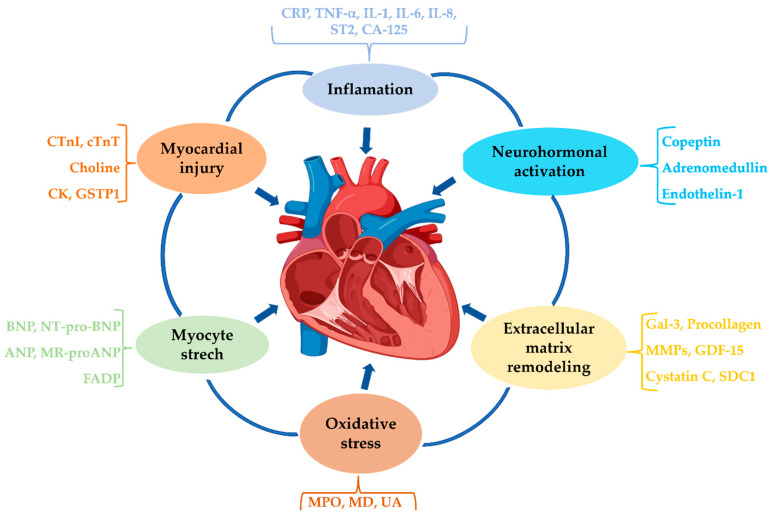
Classification of biomarkers according to the pathophysiological mechanisms involved in HF.

Given the pathophysiological mechanisms implicated in HF (inflammation, oxidative stress, cardiac remodeling, neurohormonal activation, etc.), biomarkers can be classified into several categories (Figure 2) [42,45].

### 3.1. Inflammatory Biomarkers

It is now widely accepted that inflammation plays a pivotal role in the pathogenesis and progression of HF, with the so-called *cytokine hypothesis* first proposed in 1996. Initial cardiac injury is believed to activate a cascade of pro-inflammatory cytokines which, in the early phases, exert compensatory and adaptive effects. However, persistent and excessive cytokine production shifts this response toward a maladaptive state, contributing to pathological remodeling and HF progression [46]. Several clinical studies have demonstrated that HF is associated with elevated levels of C-reactive protein, interleukins (IL-1, IL-6, IL-8), and TNF-α [42,47].

*C-reactive protein (CRP)* is a pentameric protein synthesized in the liver in response to a variety of stimuli, including IL-6. It is one of the most extensively investigated biomarkers and is regarded as an important risk factor for the development and progression of HF. CRP mediates and sustains inflammation and may accelerate cardiac remodeling. This molecule reduces nitric oxide synthesis in endothelial cells, increases endothelin-1 production, and amplifies the expression of endothelial adhesion molecules, all of which have detrimental effects on the vascular endothelium [42,48].

To enhance prognostic value, CRP measurement is recommended in conjunction with BNP levels [46]. In the presence of inflammation and tissue injury, serum CRP levels can be approximately 100-fold higher than normal values. For more accurate monitoring of chronic inflammatory processes, the high-sensitivity form of CRP (hs-CRP) is recommended. Values below 2 mg/L indicate a low cardiovascular risk, whereas higher levels (observed in more than 60% of patients with HFpE) are associated with an adverse prognosis and justify the initiation of a more aggressive therapeutic strategy [49].

Recently, the C-reactive protein (CRP)-to-albumin ratio (CAR) has been reported as a novel prognostic biomarker, proposed to be a more reliable indicator than CRP or albumin alone. Elevated CAR values are significantly associated with adverse outcomes in both acute and chronic HF, including all-cause mortality, higher NYHA functional class, increased rates of complications, organ failure, and the need for interventions, but show no association with LVEF [50].

Although the contribution of *TNF-α* to HF pathophysiology has been recognized since the 1990s, its mechanisms remain incompletely understood. In the early stages, TNF-α appears to exert a mild inotropic effect; however, with sustained inflammatory stimulation, it develops a cardiodepressant profile. While some studies have produced conflicting results, the majority confirm that TNF and its soluble receptors (sTNF-RI and sTNF-RII) are elevated in chronic HF, with equivalent prognostic value in both HFpEF and HFrEF. Furthermore, elevated TNF-α levels correlate with BNP concentrations and are associated with poor outcomes [46,49].

TNF-α and interleukins trigger multiple intracellular signaling pathways, influencing cardiac remodeling, hypertrophy, and apoptosis [42].

*Suppression of tumorigenicity 2 (ST2)*, also known as interleukin-1 receptor-like 1 (IL1RL-1), is a member of the Toll-like/IL-1 receptor (TLR) superfamily [51]. It is expressed in cardiomyocytes and is primarily upregulated in response to mechanical stress, including in the setting of hypertrophy, fibrosis, and myocardial injury, thereby contributing to worsening ventricular dysfunction [52]. ST2 exists in two isoforms: a transmembrane receptor (ST2L) and a soluble circulating receptor (sST2) detectable in serum; both share IL-33 as their sole ligand [53]. Binding of IL-33 to ST2L activates the NF-κB and MAPK signaling pathways. Experimental animal studies have shown that such activation prevents apoptosis by inducing anti-apoptotic factors, thereby improving cardiac function and increasing post-myocardial infarction survival [54].

The soluble form sST2 acts as a *decoy receptor*, binding free IL-33 and preventing its interaction with the transmembrane receptor ST2L. Unlike ST2L, sST2 lacks intracellular signaling capacity [46,54]. Tissue expression studies indicate that ST2L is predominantly expressed in cardiomyocytes, but also in a variety of immune cells, including type 2 helper T cells (Th2), regulatory T cells (Treg), polarized macrophages, mast cells, eosinophils, basophils, neutrophils, and natural killer (NK) cells [52]. In vitro studies have demonstrated that sST2 secretion can be induced by pro-inflammatory cytokines such as IL-1β and TNF-α, occurring both in cardiomyocytes and pulmonary epithelial cells. Human studies have confirmed the involvement of cardiac and pulmonary cells in sST2 production, and have also identified mast cells as a source of sST2 upon IL-33–induced activation [52].

sST2 concentrations have been demonstrated to possess significant prognostic value in both acute and chronic HF. The use of a multimarker approach incorporating sST2 and NT-proBNP enhances risk stratification and enables a more precise identification of patients at increased risk of adverse outcomes, including mortality. In contrast to BNP and NT-proBNP, the prognostic performance of sST2 is not substantially affected by common confounding factors such as obesity, advanced age, atrial fibrillation, or impaired renal function. Furthermore, evidence indicates that several therapeutic agents, particularly mineralocorticoid receptor antagonists and β-blockers, are associated with reductions in circulating sST2 concentrations [53,55].

*Cancer antigen 125 (CA 125)* is a high-molecular-weight transmembrane glycoprotein (containing approximately 22,000 amino acids) from the mucin family, also known as MUC16. Initially identified in ovarian tumors, CA 125 has subsequently been shown to be expressed in multiple epithelial structures, including the lungs, prostate, pleura, pericardium, and peritoneum. Under physiological conditions, CA 125 functions to hydrate and lubricate epithelial surfaces, protecting them from mechanical stress [56].

Elevated CA 125 levels have been reported not only in other malignancies (e.g., lung cancer, non-Hodgkin lymphoma), but also in non-malignant conditions (e.g., liver cirrhosis, pelvic inflammatory disease, peritoneal disorders, ascites), as well as in certain physiological states (e.g., pregnancy, menstruation) [57]. In HF, elevated serum levels of CA-125 have been shown to correlate with disease severity, increased cardiac filling pressures, higher risk of hospitalization and re-hospitalization for HF, as well as increased mortality [58].

The pathophysiological mechanisms underlying elevated CA 125 are multifactorial, with inflammation (via pro-inflammatory cytokines IL-6, IL-10, and TNF-α) and mechanical stress caused by fluid overload, playing major roles. Clinical studies have shown positive correlations between CA 125 levels, IL-6, and hs-CRP. Venous congestion leads to endothelial and perivascular alterations, resulting in increased concentrations of ROS, pro-inflammatory mediators, and vasoconstrictors [59].

CA 125 is also implicated in cardiac remodeling through alterations in ECM, with elevated levels correlating with ventricular dysfunction. Recent studies have highlighted the prognostic value of CA 125 in predicting all-cause mortality in HF patients, particularly within the first six months after hospital discharge. A moderate correlation has been established between CA 125 and NT-proBNP, and the combination of these two biomarkers offers improved risk stratification in HF [59].

### 3.2. Oxidative Stress Biomarkers

Myeloperoxidase (MPO) is a heme-containing enzyme from the peroxidase family, expressed by polymorphonuclear neutrophils and released during leukocyte degranulation [60]. This enzyme catalyzes the synthesis of hypochlorous acid/sodium hypochlorite and ROS, playing a key role in the process of phagocytosis. When phagosome formation is incomplete or in the presence of chronic inflammation, MPO can be released into the extracellular space. This release contributes to the degradation of essential macromolecular structures, thereby promoting the onset and progression of various disorders with an inflammatory component. Specifically, hypochlorous acid produced by MPO is a potent oxidizing agent that reacts with cellular proteins, lipids, and DNA, inducing significant structural damage that may lead to apoptosis and necrosis [46].

In addition to neutrophils, MPO is also expressed in monocytes and macrophages, accumulating at the endothelial and subendothelial levels. The presence of MPO in these sites reduces endogenous NO levels, thereby impairing vascular tone [61]. Hypochlorous acid, generated via the oxidation of Cl^−^ by H_2_O_2_ under MPO activity, can chlorinate tyrosine residues within proteins, leading to the formation of 3-chlorotyrosine, a recognized marker of oxidative stress. Consequently, MPO serves as a marker of vascular inflammation, integrating both oxidative stress and inflammatory response components [45].

Several clinical studies have demonstrated that patients with HF exhibit increased numbers of activated polymorphonuclear cells, the primary source of MPO, and consequently higher circulating MPO levels compared with healthy controls. Elevated MPO concentrations have been observed in patients with acute and chronic HF, and showing a positive correlation with BNP concentrations [46]. It has been reported that MPO concentrations above 99 pmol/L are significantly associated with increased 1-year mortality in patients with acute HF (HR 1.58; *p* = 0.02), with prognostic accuracy further improved when MPO and BNP are combined (HR 2.80; *p* < 0.001). Moreover, plasma MPO levels increase in accordance with NYHA class and remain strongly correlated with plasma BNP concentrations [53].

### 3.3. Cardiac Remodeling Biomarkers

*Galectin-3 (Gal-3)* is a 30 kDa glycoprotein belonging to the β-galactoside-binding lectin family. It has been identified in fibroblasts and macrophages and is involved in cell adhesion, activation, proliferation, apoptosis, and migration [46]. Gal-3 plays a key role in both acute and chronic inflammation, as well as in the induction of fibrosis in various tissues and organs, including the myocardium, kidneys, and liver [62].

Administration of Gal-3 into the pericardium of healthy rats has been shown to induce cardiac fibroblast proliferation, stimulate collagen synthesis and deposition, and impair left ventricular function. In clinical studies, elevated Gal-3 levels have been correlated with echocardiographic parameters of ventricular dysfunction and have demonstrated prognostic value for major adverse cardiovascular events, such as death and hospitalizations, particularly in patients with HFpEF [54]. Moreover, inhibition of Gal-3 expression at the cardiac level has been shown to slow fibrosis progression and improve ventricular remodeling. While the diagnostic utility of Gal-3 is lower than that of NT-proBNP, its prognostic relevance remains significant, especially in the context of HFpEF [46,49].

*Procollagen*. Collagen synthesis is known to play an important role in myocardial tissue remodeling following acute MI and in HF development. Consequently, markers of collagen synthesis and turnover are used as non-invasive methods for assessing the extent of myocardial fibrosis [62]. The amino-terminal propeptide of type III procollagen (PIIINP) is one of the most studied fibrosis markers, with elevated levels reported in acute MI, HF, and chronic kidney disease [39]. High PIIINP concentrations have been shown to predict left ventricular dilatation and the development of HF within one-year post-MI; in chronic HF, PIIINP has also been identified as a predictor of mortality [46,49].

Matrix metalloproteinases and tissue inhibitors of metalloproteinases

Matrix metalloproteinases (MMPs) are zinc-dependent endopeptidases involved in the degradation of ECM proteins, as well as other cellular proteins. Elevated MMP levels are associated with inflammation, acute MI, and ventricular dysfunction in HF [49]. MMPs are inhibited by a class of molecules known as tissue inhibitors of metalloproteinases (TIMPs) [52]. An imbalance between MMPs and TIMPs, characterized by increased MMP activity, is believed to play an important role in ventricular remodeling [62]. MMPs and TIMPs have been extensively studied in cardiac disease, particularly in MI and HF. Recent studies have reported that, in acute MI, elevated MMP-3 and TIMP-1 levels predict LVEF and, along with MMP-9, are associated with an adverse prognosis. Elevated MMP-2 concentrations have also been linked to hemodynamic impairment and may have prognostic value for pulmonary vascular disease and HF [46,49].

*Growth differentiation factor-15 (GDF-15)* is a member of the TGF-β superfamily, acting as a stress-responsive cytokine and associated with cardiometabolic risk [51,63]. In healthy individuals, GDF-15 expression is restricted to the central nervous system and placenta; however, under pathological conditions involving injury, hypoxia, oxidative stress, or inflammation, it is strongly expressed in cardiomyocytes, adipocytes, macrophages, endothelial cells, and vascular smooth muscle cells [64,65].

Although the molecular mechanisms of action are not fully elucidated, studies suggest a potential protective role through inhibition of pro-apoptotic molecules (JNK, Bcl-2-associated death promoter, epidermal growth factor receptor) and activation of pro-survival signaling pathways (Smad, eNOS, PI3K, and AKT) [45]. GDF-15 expression is upregulated in cardiomyocytes following ischemia/reperfusion injury and MI, particularly in the peri-infarct zone, where it appears to exert cardioprotective effects. GDF-15 was reported to be a useful prognostic biomarker for mortality in patients with HF or MI as well as for thrombosis among patients with atrial fibrillation [66]. Animal studies have shown that GDF-15 is overexpressed in MI following exposure to oxidized LDL, and thus also in atherosclerotic coronary artery disease [46]. Elevated GDF-15 concentrations have been associated with HF progression, and the two-year mortality risk is four times higher in patients with elevated levels of both GDF-15 and NT-proBNP. Consequently, the long-term prognostic value of GDF-15 in HF is enhanced when combined with NT-proBNP measurements [49]. Unlike classical biomarkers such as NT-proBNP, which are significantly higher in HFrEF compared with HFpEF, GDF-15 levels have been found to increase similarly in both HF phenotypes [67].

In patients with ischemic and non-ischemic cardiomyopathies undergoing primary prevention implantable cardioverter-defibrillator therapy, elevated levels of GDF-15 have been associated with an increased risk of HF hospitalization and all-cause mortality, but not with ventricular arrhythmic events [66]. Higher circulating GDF-15 levels have also been correlated with elevated cardiac filling pressures, increased LV mass, and impaired LV systolic function [68]. Furthermore, in advanced HFrEF, increased plasma GDF-15 levels have been linked to a greater prevalence of anorexia and cachexia, right ventricular dysfunction, and systemic congestion, and independently predict a higher risk of adverse clinical outcomes [69].

*Cystatin C* is a cysteine protease inhibitor involved in the regulation of vascular cathepsins S and K. It is produced at a constant rate by all nucleated cells, independent of inflammatory, infectious, or neoplastic processes, and is not influenced by other factors such as muscle mass, sex, age, diet, or concomitant medication [61]. Due to its small molecular size, cystatin C is freely filtered by the glomeruli and reabsorbed in the proximal tubules, making it a useful marker for estimating glomerular filtration rate (GFR) and, consequently, renal function [70]. It provides a more accurate assessment of renal function in HF patients than serum creatinine and has demonstrated superior prognostic value [71].

Given its involvement in ECM remodeling, cystatin C is considered to have additional value beyond GFR estimation, being associated with structural and functional parameters of the left ventricle, unlike creatinine. Studies have shown a strong correlation between cystatin C and the risk of major cardiovascular events, including stroke, MI, and cardiovascular death. Furthermore, cystatin C levels have been shown to correlate significantly with systolic dysfunction in HF patients [72].

### 3.4. Myocardial Stress/Hemodynamic Biomarkers

The inability of the heart to pump blood at a rate sufficient to meet the body’s metabolic demands results in increased hemodynamic stress, primarily through sodium and water retention at the renal level [45]. At the cardiac level, pressure or volume overload leads to increased concentrations of ANP and BNP as compensatory mechanisms [9,73]. *Natriuretic peptides* (NPs) exert inhibitory effects on the sympathetic nervous system and the RAAS while promoting diuresis, natriuresis, reduced peripheral vascular resistance, and relaxation of vascular smooth muscle with consequent vasodilation [62].

**Figure 3:**
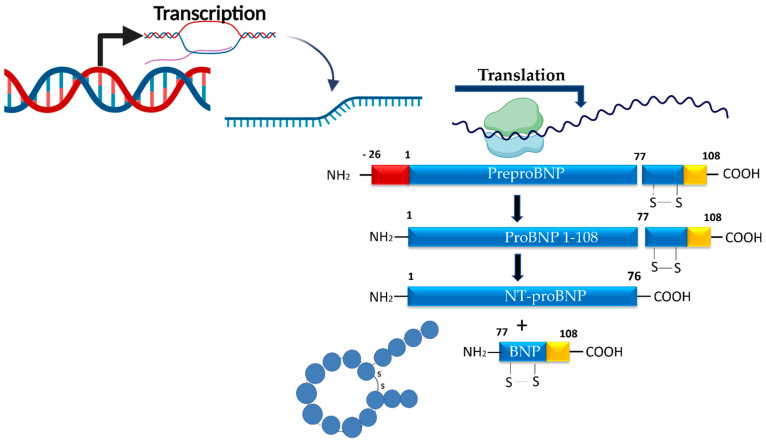
Synthesis pathways of BNP and NT-proBNP from proBNP at the myocardial level.

The synthesis of NPs is directly linked to hemodynamic stress, which activates the myocardial BNP gene, rapidly stimulating mRNA transcription, followed by translation into the prohormone proBNP1–108. This prohormone is subsequently cleaved into the biologically active C-terminal fragment (BNP, consisting of 32 amino acids) and the biologically inactive N-terminal fragment (NT-proBNP, consisting of 76 amino acids), which is considerably more stable than BNP [61,62]. BNP contains a central ring of 17 amino acids stabilized by a disulfide bond between two cysteine residues (Figure 3).

NPs exert their effects through specific membrane-bound NPs receptors (NPRs), of which three subtypes are known: NPR-A, NPR-B, and NPR-C. Activation of the main signaling receptors, NPR-A and NPR-B, stimulates intracellular cyclic guanosine monophosphate (cGMP) production, which mediates physiological effects such as diuresis, natriuresis, vasodilation, and attenuation of cardiac hypertrophy and fibrosis. In contrast, NPR-C functions primarily as a clearance receptor, facilitating the degradation of NPs. Furthermore, the synthesis of NPs can be induced by ischemia, hypoxia, and various neurohumoral mediators [45,53].

The half-life of BNP is approximately 20 min, as it is rapidly degraded by plasma endopeptidases, primarily neprilysin. In contrast, NT-proBNP is metabolized renally and has a longer half-life of 60–120 min, resulting in serum concentrations roughly six times higher than those of BNP, despite both molecules being released simultaneously in equimolar amounts [45,64].

In the case of ANP, its short half-life of only 2–5 min limits its diagnostic utility. This disadvantage has led to the clinical introduction of its prohormone, proANP, as a more relevant biomarker, given its longer half-life and greater stability compared to ANP. Serum quantification of proANP is performed by measuring the mid-regional pro-atrial natriuretic peptide fragment (MR-proANP), whose prognostic value for estimating mortality risk in chronic HF is comparable to that of NT-proBNP [45,47].

Current guidelines recommend BNP and its precursor, NT-proBNP, as the most important biomarkers for the diagnosis and monitoring of HF [9]. Although circulating concentrations of BNP and NT-proBNP are typically low in healthy adults, with slightly higher values reported in women compared to men (e.g., approximately 14 pg/mL vs. 8 pg/mL), their levels increase substantially in the context of HF, reflecting elevated myocardial wall stress and impaired cardiac function [53].

NPs also have a significant prognostic role in HF, serving as powerful predictors for both cardiac mortality and all-cause mortality. They can also identify adverse prognostic elements, such as severe non-fatal cardiac events or HF-related rehospitalizations [45].

Furthermore, plasma BNP levels have been shown to increase in parallel with HF severity, with a demonstrated correlation between NPs concentrations and the NYHA functional class [54]. It has been reported that every 100 pg/mL increase in BNP is associated with a 35% higher relative risk of death. However, it should be taken into account that several factors, including advanced age, female sex, renal dysfunction, and treatment with sacubitril/valsartan, can elevate BNP levels even in the absence of HF [52].

In addition, other authors have reported that NPs independently predict the presence of HFpEF or asymptomatic HF, with these diagnoses later confirmed by echocardiography [64].

In chronic HF, some studies have demonstrated that MR-proANP has a superior prognostic value compared with NT-proBNP for predicting mortality. Moreover, combining these two biomarkers appears to improve both diagnostic accuracy and prognostic performance in this patient population [45,60].

While most studies indicate that all NPs have similar diagnostic value, there is also evidence supporting a superior long-term prognostic value for MR-proANP in patients with acute HF [45].

**Figure 4:**
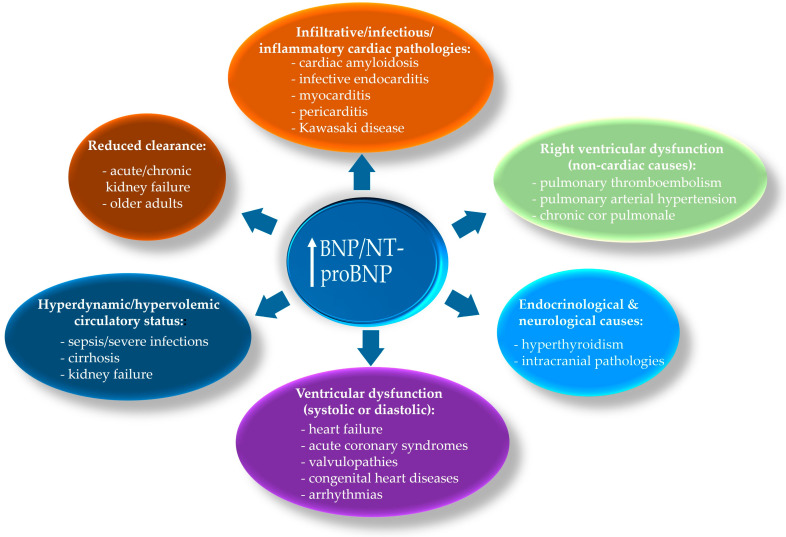
Conditions associated with elevated BNP and NT-proBNP levels.

Although NPs are highly sensitive biomarkers for HF diagnosis, their specificity is limited. Elevated concentrations may also occur in other conditions that directly or indirectly affect cardiac performance. Furthermore, such variations can arise even in the absence of myocardial dysfunction, being influenced by non-cardiac mechanisms, some of which are constitutional and non-modifiable (e.g., age) (Figure 4).

Chronic kidney disease (CKD) is among the most frequently implicated comorbidities in reducing the diagnostic accuracy of NT-proBNP, being commonly encountered in clinical practice and often associated with HF. Studies have shown that NT-proBNP has the highest diagnostic sensitivity in patients with a GFR greater than 90 mL/min/1.73 m^2^, with this performance being markedly diminished in patients with advanced renal dysfunction and a GFR below 30 mL/min/1.73 m^2^. In contrast, due to its partial renal clearance, BNP levels are less influenced by renal function, making it more suitable for assessing cardiac function in patients with moderate-to-severe renal impairment. Moreover, elevated NT-proBNP levels have been associated with a 2–4-fold higher risk of sudden cardiac death or stroke compared with normal or mildly elevated values [53,64].

Infectious pathology represents another major non-cardiac cause of elevated NP levels. Systolic dysfunction and cardiac chamber dilatation are well-documented phenomena in the context of sepsis, particularly in severe forms progressing to septic shock. Increased BNP/NT-proBNP concentrations have also been reported in patients with common respiratory tract infections, especially in those with DM. A possible explanation may lie in the fact that tissue hypoxia associated with infectious states induces microlesions at the cardiac level, leading to the subsequent release of NPs into the circulation [74]. Furthermore, in severe sepsis, NT-proBNP has been shown to be a prognostic marker for mortality, with serum levels measured at admission and at 72 h being significantly higher in non-survivors compared with survivors [54].

*Soluble neprilysin (sNEP)*. Neprilysin (NEP) is a transmembrane zinc-dependent metalloendopeptidase with a molecular weight of approximately 90 kDa. It consists of a large extracellular catalytic domain, a single transmembrane region, and a short cytoplasmic N-terminal domain of 27 amino acids [75]. NEP is highly expressed in renal proximal tubules, but it is also present in the lungs, vascular endothelium, vascular smooth muscle cells, cardiomyocytes, fibroblasts, neutrophils, adipocytes, testes, and the brain [76]. Functionally, NEP catalyzes the degradation of several vasoactive and vasodilatory peptides, including NPs, Ang II, bradykinin, substance P, adrenomedullin, and endothelin-1. It is estimated that NEP is responsible for the clearance of at least 50% of circulating NPs, thereby modulating their antifibrotic, antiproliferative, vasodilatory, and myocardial relaxation effects. Like other membrane-bound metalloproteases, NEP can be shed from the cell surface, generating a soluble form (sNEP) that circulates in plasma and urine while retaining full enzymatic activity [77].

Several studies have demonstrated the prognostic value of sNEP in HF. High sNEP levels have been associated with an increased risk of cardiovascular death, acute decompensated HF, and recurrent all-cause or cardiovascular hospitalizations in chronic HF patients [75,76]. In a study of 1021 ambulatory HF patients followed for a median of 3.4 years, elevated sNEP levels independently predicted repeated HF hospitalizations and cardiovascular mortality [75]. The prognostic relevance of sNEP, however, appears to differ across HF phenotypes. In HFrEF, elevated sNEP levels were strongly associated with adverse outcomes, supporting the concept that NEP inhibition may be beneficial in this population [76]. Conversely, in HFpEF, sNEP levels did not correlate with NYHA functional class, 6-min walk distance, hospitalization, or mortality. Moreover, patients with HFpEF were found to have lower sNEP concentrations compared with controls without diastolic dysfunction or HF, suggesting distinct pathophysiological mechanisms [77].

An additional strength of sNEP as a biomarker lies in its relative independence from comorbidities that influence other markers such as NT-proBNP. For instance, in patients with CKD stages 2–4, reduced sNEP activity (but not concentration) was predictive of future HF hospitalization, highlighting the potential added value of measuring enzymatic activity rather than concentration alone [78].

*Fatty acid-binding proteins (FABPs)* are intracellular proteins composed of 126–137 amino acids, with a molecular weight ranging from 15 to 20 kDa. FABPs are predominantly found in tissues with high rates of fatty acid metabolism, including the intestine, liver, and heart, and exhibit high affinity for lipids [51,79]. FABPs are encoded by different genes, and their nomenclature reflects their tissue-specific expression. For example, H-FABP (heart-type fatty acid-binding protein) is found primarily in cardiomyocytes but is also present in skeletal muscle and distal convoluted tubule cells. In the setting of myocardial injury, H-FABP becomes detectable in the circulation as early as approximately 20 min after the acute event, reaching peak serum concentrations within 3–4 h and returning to baseline values within 18–30 h [45,51].

Studies have demonstrated both the diagnostic and prognostic value of H-FABP in acute and chronic HF, with elevated levels being associated with disease progression through worsening cardiac dysfunction, frequent hospitalizations, and an increased risk of death. Additionally, in chronic HF, the combination of H-FABP with BNP has proven useful for risk stratification regarding mortality and non-fatal cardiovascular events [45].

### 3.5. Biomarkers of Neurohormonal Activation

**Figure 5:**
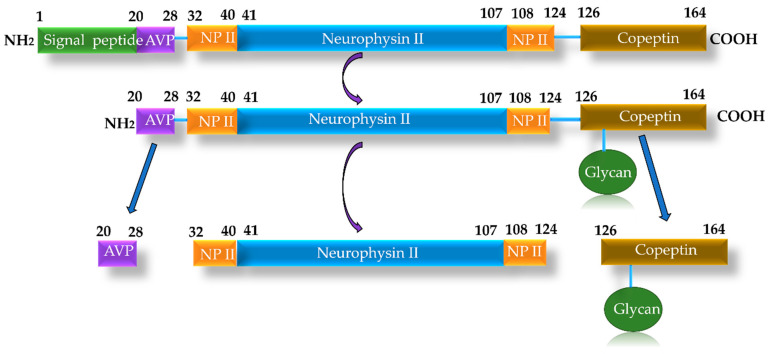
Synthesis pathway of copeptin from pre-pro-vasopressin.

Copeptin (CT-proAVP) is a glycopeptide composed of 39 amino acids, with a molecular mass of 5 kDa. It results from the enzymatic cleavage of the pre-pro-hormone pro-vasopressin, forming its C-terminal segment, along with vasopressin and neurophysin, which are released into the circulation in equimolar amounts [79,80] (Figure 5). Vasopressin, also known as antidiuretic hormone (ADH) or arginine vasopressin, is a peptide hormone synthesized in the hypothalamus and stored in the posterior lobe of the pituitary gland. ADH exerts both antidiuretic and vasoconstrictive effects, with elevated levels frequently observed in patients with HF. Excessive ADH secretion can lead to hyponatremia, edema, and systemic vasoconstriction.

Unfortunately, ADH is difficult to quantify, as it exhibits a high degree of binding to plasma proteins and has a half-life of only 24 min. In contrast, the C-terminal fragment of pre-pro-vasopressin, copeptin, is highly stable, with a half-life of several days. It can be easily measured and correlates strongly with ADH levels [81]. In both serum and plasma samples, copeptin remains stable for at least 7 days at room temperature and 14 days at 4 °C, and can be readily quantified using the ELISA method. For quantification, copeptin requires only 50 µL of plasma, whereas several milliliters are needed for ADH measurement [81].

In the BACH (Biomarkers in Acute Heart Failure) study, patients with elevated copeptin concentrations presented with more severe pulmonary and peripheral congestion, as well as an increased 3-month mortality rate [61]. In another study, a meta-analysis including 4473 patients with acute and chronic HF, copeptin proved to be a reliable prognostic marker for all-cause mortality, with a performance comparable to that of NT-proBNP [61].

*Adrenomedullin (ADM)* is a low-molecular-weight peptide hormone (6 kDa) synthesized in almost all tissues, but predominantly in the adrenal medulla, heart, lungs, and kidneys, in response to volume and pressure overload [70,79]. ADM exerts vasodilatory, natriuretic, inotropic, and cardioprotective effects, while also maintaining endothelial barrier integrity. In cases of ADM deficiency, increased membrane permeability occurs, leading to generalized edema or even pulmonary congestion and acute pulmonary edema [61].

Under physiological conditions, serum ADM concentrations are low (approximately 13 pg/mL) but increase three- to four-fold in patients with HF, exerting beneficial effects through inhibition of the RAAS [70]. Similar to ADH, plasma ADM quantification is challenging due to its short half-life and high degree of binding to plasma proteins. Measurement is facilitated for a fragment of the ADM precursor, namely, MR-proADM (mid-regional fragment of pro-adrenomedullin), which has been shown to outperform NT-proBNP and BNP in predicting 90-day survival in patients with acute HF and dyspnea, according to data from clinical trials such as BACH and PRIDE [53,61].

The prognostic value of MR-proADM has also been demonstrated in the context of chronic HF. A study including 501 patients with chronic HF showed that its levels correlated comparably to NT-proBNP in predicting one-year survival. Nevertheless, the clinical utility of MR-proADM is limited by the fact that it is not specific for cardiac injury, being expressed in numerous tissues and influenced by a wide range of pathophysiological factors [61].

### 3.6. Biomarkers of Cardiomyocyte Injury

Troponins (Tns) are a protein complex located within the sarcomere, itself composed of actin and myosin filaments. The complex consists of three regulatory subunits, C (TnC), T (TnT), and I (TnI), each playing distinct roles in muscle contraction and actin–myosin interaction [62]. During the excitation phase, TnC binds calcium ions, and the resulting complex interacts with tropomyosin, unblocking the active sites on actin and myosin filaments, thereby enabling their interaction and myofibrillar contraction (systole). This effect diminishes as extracellular calcium concentration decreases due to intracellular uptake. Phosphorylation of TnI prevents calcium binding to TnC, and the released tropomyosin subsequently blocks actin–myosin interaction, resulting in myofibrillar relaxation (diastole) [60,61]. While TnC is found in both cardiac and skeletal muscle fibers, TnI and TnT are specific to cardiac muscle. Given this tissue-specific distribution, only TnI and TnT have value as biomarkers in cardiac disorders [43,45].

Within cardiomyocytes, Tn exists in two forms: a cytosolic, functionally active pool, and a predominant sarcomeric, structurally bound pool. Prolonged ischemia induces myocyte injury, progressing to necrosis, with disruption of the cell membrane and subsequent release of Tn into the circulation in distinct patterns. The sarcomeric fraction is released slowly over several days to up to two weeks, whereas the cytosolic fraction is released rapidly, within 4–6 h of injury onset [45,70].

Increases in Tn levels (cTnI ~ 1.0 mg/mL, cTnT ~ 0.1 mg/mL) correlate with the extent of cellular damage, peaking during ischemic myocardial necrosis. Consequently, elevated serum Tn levels represent a key diagnostic element in acute MI. Tns, particularly TnI, have been detected not only in acute coronary syndromes but also in non-ischemic myocardial injuries, such as myocarditis, drug-induced cardiotoxicity, and multifactorial conditions including HF (with or without an ischemic component), pulmonary embolism, and stress cardiomyopathy [53,82].

In HF, plasma Tn concentrations have shown significant correlation with LVEF. Myocardial injury through apoptosis and necrosis contributes to increased mechanical stress on the heart, leading to progressive reductions in myocardial perfusion and oxygenation. Furthermore, impaired renal clearance exacerbates these hemodynamic and metabolic disturbances [45].

Although Tns have been primarily used in the diagnosis of both acute and chronic HF, their prognostic value has also been reported. For example, the ADHERE (Acute Decompensated Heart Failure National Registry) study identified an increased mortality risk in patients with elevated plasma Tn levels [45,62].

In chronic HF, measurement of Tn can be challenging, which has led to the development of high-sensitivity assays (hs-cTn) capable of detecting extremely low concentrations, particularly of the cytosolic fraction. These assays offer superior sensitivity, enabling quantification of concentrations 10–100 times lower than with conventional methods [62].

Elevated Tn levels in acute HF have been associated with a significantly increased risk of all-cause mortality at one year, independent of the presence or absence of acute ischemia. Another study demonstrated that elevated plasma TnI at hospital admission was associated with lower LVEF, higher estimated pulmonary artery pressure, prolonged hospitalization, and increased long-term mortality [83]. Notably, elevated Tn levels have been linked to higher all-cause, cardiac, and non-cardiac mortality [52,84]. However, in the setting of chronic HF, elevated Tn levels alone are insufficient to effectively guide therapeutic strategies; instead, an integrated approach using multiple biomarkers, including NT-proBNP and other complementary markers, is required [45,83].

*Glutathione S-transferase P1 (GSTP1)* is the most important enzyme in the glutathione S-transferase (GST) family, playing a critical role in cellular detoxification and protection against oxidative stress. GSTP1 is an endogenous inhibitor of c-Jun N-terminal kinase (JNK), a pro-apoptotic enzyme involved in the MAPK signaling pathway. It also acts as an endogenous inhibitor of tumor necrosis factor receptor–associated factor 2 (TRAF2), thereby suppressing TRAF2-induced MAPK activation. GSTP1 expression is upregulated as an adaptive response to oxidative stress and pro-inflammatory stimuli, both of which are frequently implicated in the pathophysiology of chronic HF. This supports the hypothesis that GSTP1 may be associated with advanced stages of the disease. In particular, patients in NYHA functional classes III–IV have shown elevated levels of both GSTP1 and NT-proBNP; however, the diagnostic value of GSTP1 was found to be superior to that of NT-proBNP [45].

*Choline* is released into the circulation following phospholipid cleavage from the cell membrane in response to ischemic injury. In combination with Tn, choline serves as a biomarker with significant prognostic value for identifying the risk of major cardiovascular events, cardiac arrest, and death. It is frequently used for risk stratification in patients with elevated troponin levels [60].

*Creatine kinase (CK)* catalyzes both the synthesis of phosphocreatine from creatine in the presence of ATP and the regeneration of ATP from ADP and phosphocreatine. CK is found in higher concentrations in skeletal muscle (CK-MM) and myocardium (CK-MB) and in lower concentrations in the brain (CK-BB) [79]. The myocardium contains approximately 30% CK-MB, which limits the specificity of this enzyme for cardiac disorders. Although the sensitivity and specificity of CK-MB for myocardial infarction diagnosis are lower than those of Tn, this biomarker remains useful in specific clinical contexts, particularly for detecting reinfarction. Elevated CK-MB levels are typically detectable within 3–4 h after the onset of acute MI, reach peak concentrations within approximately 24 h, and return to baseline within 24–72 h. Physiologically, the CK-MB/CK ratio is approximately 1, and elevated values of 2.5–3 suggest myocardial injury. However, elevated CK-MB levels may also occur in other conditions, such as chest trauma or substance use (e.g., cocaine), necessitating careful clinical correlation [60,79].

### 3.7. Other Types of Biomarkers

*Syndecans (SDCs)* are proteins belonging to the large family of proteoglycans, acting as co-receptors for G-protein-coupled receptors. They are expressed in both the peripheral and central nervous systems and are primarily involved in cell adhesion, migration, and growth factor signaling [85]. All four members, SDC1 to SDC4, share a similar structure, consisting of an extended extracellular domain containing glycosaminoglycan attachment sites, a transmembrane domain, and a smaller cytosolic domain [86]. SDC3 is predominantly expressed in the central nervous system, particularly within axons and synapses, and is therefore also referred to as neural syndecan (N-SDC). In contrast, SDC2 and SDC4 are highly expressed in astrocytes, with lower expression in neurons [87]. SDC1 functions as a receptor for ECM components, with affinity for collagen and fibronectin, and has been linked to cardiac fibrosis [85].

Several experimental studies have reported a direct correlation between serum SDC1 and NT-proBNP levels, as well as with various adverse prognostic factors in HF, including blood pressure, reduced LVEF, and higher rates of rehospitalization. Its prognostic value is enhanced when used in combination with other biomarkers in both HFpEF and HFrEF. Regarding its role in HF pathogenesis, SDC1 has been implicated in cardiac fibrosis, atherogenesis, and neurohormonal activation, particularly through the RAAS [88]. The profibrotic effects of Ang II and associated cardiac remodeling occur via concurrent activation of TGFβ1 and connective tissue growth factor (CTGF), resulting in increased synthesis of collagen and other ECM proteins [89].

Animal models of HF have shown marked SDC1 expression in injured tissues, implicating it in both inflammatory and reparative processes. Clinical studies have demonstrated that elevated SDC1 levels are associated with fibrosis and cardiac remodeling markers, as well as impaired renal function. In HFpEF, higher SDC1 concentrations have been linked to increased all-cause mortality and higher rehospitalization rates. However, these associations have not been confirmed in HFrEF, and its role in other HF subtypes, such as those secondary to cardiomyopathies, remains insufficiently studied [90].

*Insulin-like Growth Factor–Binding Protein 7 (IGFBP7)* belongs to the family of proteins with affinity for insulin-like growth factors (IGFs). It binds both IGF-1 and insulin, with a 500-fold higher affinity for insulin. This interaction inhibits insulin binding to its receptor, thereby reducing physiological insulin responses. Such a mechanism contributes to insulin resistance, favoring the development of T2DM and associated cardiovascular complications [91].

In addition, IGFBP7 is associated with cellular senescence-promoting processes, including inhibition of cell division, increased oxidative stress, impaired DNA repair, and promotion of fibrosis. This protein is a component of the senescent cell secretome, with its effects seemingly mediated through interference with the protective functions of the transcription factor FOXO3a, known for reducing cellular stress and promoting autophagy [92]. Given its involvement in cellular growth, proliferation, and differentiation, IGFBP7 is considered a marker of cellular senescence and tissue aging [93].

IGFBP7 is also of interest in various cardiovascular and non-cardiovascular diseases, such as atherosclerosis, atrial fibrillation, DM, malignancies, HF, and CKD [78]. In HFpEF, elevated IGFBP7 levels have been associated with left atrial remodeling and diastolic dysfunction. Similar findings were reported in a large epidemiological study including patients aged 65–84 years with left ventricular hypertrophy and atrial fibrillation [94]. Based on these observations, IGFBP7 has been proposed as a biomarker reflecting premature tissue aging and myocardial fibrosis, changes that contribute to diastolic dysfunction. Its diagnostic and prognostic value is enhanced when combined with NT-proBNP in both acute and chronic HF [92].

*Endothelins (ETs)* are vasoactive peptides composed of 21 amino acids. Three isoforms are known, ET-1, ET-2, and ET-3, each encoded by distinct genes. Under physiological conditions, ET-1 is produced by vascular endothelial cells, acting as a local paracrine and autocrine mediator [86]. In certain pathological states, ET-1 expression also occurs in vascular smooth muscle cells, cardiomyocytes, and inflammatory cells. The biological effects of ET-1 are mediated by two receptors with opposing actions: ETA receptors promote vasoconstriction and inflammation, while ETB receptors mediate vasodilation, natriuresis, and anti-inflammatory effects [53].

ET-1 is notably associated with increased pulmonary vascular pressure, a phenomenon observed in both HF and idiopathic pulmonary hypertension. Through persistent vasoconstriction of the pulmonary arteries, ET-1 increases pulmonary vascular resistance, eventually leading to pulmonary hypertension and vascular remodeling. These changes may secondarily increase right atrial and right ventricular pressures, with hemodynamic consequences on global cardiac function [95].

Elevated ET-1 levels have been associated with increased mortality and echocardiographic parameters suggestive of HF, such as left atrial volume and left ventricular hypertrophy. In HFpEF, high ET-1 levels have been linked to long-term mortality and increased HF-related hospitalizations [96].

*Trimethylamine N-oxide (TMAO)* has emerged as a biomarker within the “intestinal hypothesis” of HF, which postulates a role of the gastrointestinal tract in HF pathophysiology. TMAO is an endogenous metabolite produced from choline and carnitine by gut microbiota, representing a potential link between a Western diet and cardiovascular risk. A growing body of evidence supports the prognostic utility of TMAO in both acute and chronic HF, with significant geographical and ethnic variations. In HFpEF, its prognostic value is enhanced when combined with NPs (BNP and NT-proBNP). Despite consistent supporting evidence, TMAO is not yet included among guideline-recommended biomarkers for HF management [54,97].

*Proenkephalin (PENK)* is a 243-amino acid precursor of endogenous opioid peptides. It is associated with HF through sympathetic nervous system activation, suggesting a potential role in disease pathophysiology and prognosis. Direct enkephalin measurement is challenging due to their short half-life; however, the enzymatic degradation fragment PENK 119–159 is stable in plasma and cerebrospinal fluid for at least 48 h, serving as a surrogate marker of systemic enkephalin levels. Elevated PENK levels have been associated with reduced LVEF and impaired renal function, correlating with adverse outcomes in both acute and chronic HFrEF. High PENK concentrations have also been linked to body mass index, diastolic dysfunction, and HFpEF prognosis. These findings suggest PENK as a valuable biomarker for assessing combined cardiac and renal dysfunction, highlighting the intrinsic link between HF and the opioid system [54].

*MicroRNAs (miRNAs)* are small non-coding RNA molecules, 18–25 nucleotides in length, that inhibit messenger RNA (mRNA) translation and/or promote its degradation. More than 1000 miRNAs have been identified, many of which are abundant in blood and detectable in plasma, platelets, erythrocytes, and nucleated blood cells [98]. They play essential roles in pathological processes as cellular response mediators to various stressors [99]. Altered expression of circulating miRNAs has been linked to multiple diseases, including cancer, neurodegenerative disorders, atherosclerosis, obesity, DM, and cardiovascular disease [54].

Circulating miRNA profiles reflect tissue-specific expression changes or altered intercellular communication, suggesting their potential use as biomarkers. In angiogenic cells isolated from patients with chronic HF, reduced levels of miR-126 and miR-130a have been correlated with cardiac dysfunction. Furthermore, miR-126 and miR-508-5p expression in endothelial progenitor cells has demonstrated prognostic value in chronic HF [54].

Small pilot studies have shown that miR-423-5p has significant diagnostic value in HF, being correlated with NT-proBNP levels and strongly associated with HF diagnosis at hospital admission. Moreover, its expression is closely related to disease severity, including left ventricular ejection fraction and NYHA functional class [98].

Additional studies indicated that circulating miRNAs, particularly miR-499-5p and miR-423-5p, change dynamically in response to therapy, highlighting their potential use as biomarkers for monitoring treatment response [54]. Furthermore, a recent meta-analysis demonstrated that reduced levels of several miRNAs, including miR-30, miR-423-5p, and miR-18, are associated with poorer overall survival, highlighting their prognostic relevance in HF [100].

*Exosomes* are nanosized extracellular vesicles (30–150 nm) released by various cell types, including those of the cardiovascular system, and are present in multiple body fluids such as blood, urine, and saliva. They originate through exocytosis and carry bioactive molecules (including DNA, mRNAs, miRNAs, long non-coding RNAs, proteins, and metabolites), making them key mediators of intercellular communication [101]. In HF, distinct exosomal miRNA signatures have been associated with specific disease phenotypes and comorbidities. In patients with HFrEF, circulating exosomal miR-92b-5p has been correlated with impaired LV systolic function and chamber dilation, while miR-425 and miR-744 have been implicated in the development of cardiac fibrosis. In contrast, in HFpEF, exosomal miR-30d-5p and miR-126a-5p have been proposed as potential non-invasive diagnostic biomarkers. Furthermore, in comorbid HF conditions, altered exosomal miRNA profiles have been described: elevated miR-27a-5p combined with decreased miR-139-3p has been linked to chronic HF with hyperuricemia, whereas miR-144-3p has been associated with HF complicated by depression. These findings underscore the potential of exosomal miRNAs as disease-specific biomarkers, offering novel insights into pathophysiology and opportunities for precision diagnostics in HF [102,103].

*Fibroblast Growth Factor 23 (FGF23)* is involved in phosphate and vitamin D metabolism and regulation, playing a significant role in cardiorenal risk stratification. Secreted by osteocytes in response to increased calcitriol levels, its main function is to reduce plasma phosphate by downregulating sodium–phosphate cotransporter NPT2 expression in the proximal renal tubules, thereby decreasing phosphate reabsorption and increasing its excretion. Elevated FGF23 levels have been associated with severe HFrEF, poor prognosis, and significant renal impairment. It has also shown promise as a prognostic biomarker in HFpEF [54,104].

FGF23 plays a key role in initiating myocardial remodeling and the progression of cardiac dysfunction, offering insights into the link between phosphate homeostasis, primarily regulated by the skeletal system, and cardiac remodeling and HF pathophysiology. In a study including 120 patients with HFrEF (NYHA class II–IV), intact FGF23 (iFGF23) was shown to be an important predictor of cardiovascular mortality, improving the predictive capacity of conventional biomarkers such as NT-proBNP [104].

*Soluble Urokinase Plasminogen Activator Receptor (suPAR)* is the circulating form of uPAR, a protein receptor composed of three domains (DI, DII, and DIII) anchored to glycosylphosphatidylinositol (GPI). uPAR is encoded by the *PLAUR* (Plasminogen Activator, Urokinase Receptor) gene and is synthesized by various immune cells. suPAR is generated by proteolytic cleavage of uPAR in response to inflammatory stimuli and has been associated with poor prognosis in several conditions, including HIV infection, cancer, cardiovascular disease, T2DM and CKD. In HF, suPAR levels have been shown to correlate with NT-proBNP concentrations. Experimental models have also implicated uPAR in TGFα-1 modulation, post-infarction fibrosis, and apoptosis regulation, suggesting a direct role of suPAR in HF pathogenesis [51,105].

*Irisin* is a type I transmembrane glycoprotein initially described as a hormone secreted by muscle cells via overexpression of peroxisome proliferator–activated receptor gamma coactivator 1-alpha (PGC-1α). This overexpression activates the fibronectin type III domain-containing protein 5 (FNDC5) gene, the precursor of irisin, which is then cleaved to generate the active hormone [106].

Although initially believed to be muscle-derived, immunohistochemical studies have shown that irisin is also present in other tissues, such as the testes, stomach, and heart. Its main functions in these organs include stimulating thermogenesis, mitochondrial energy metabolism, fatty acid metabolism, and glucose utilization. Early research focused on metabolic diseases, but interest has since expanded to cardiovascular disorders [107].

The relationship between irisin and cardiovascular disease has been explored in both acute (myocardial infarction, ischemia–reperfusion injury, acute myopericarditis, acute HF) and chronic conditions (coronary artery disease, cardiac hypertrophy, chronic HF). Its cardioprotective effects include improved cardiac remodeling, enhanced cardiomyocyte survival, increased intracellular calcium concentrations, and reduced pro-inflammatory mediator levels [106].

In patients with coronary atherosclerosis, elevated irisin levels have been associated with adverse cardiovascular events, while acute HF patients with high irisin concentrations have shown increased one-year mortality risk. This high mortality rate may be explained by increased ATP consumption stimulated by irisin, which is released via a maladaptive compensatory mechanism [108].

While the prognostic value of irisin in acute HF is well established, data in chronic HF are contradictory. In animal models of cardiac hypertrophy, reduced irisin concentrations have been observed, and myokine treatment improved volume overload-induced hypertrophy. Similarly, in chronic HF animal models, administration of TNF-α and Ang II reduced FNDC5 levels and PGC-1α mRNA expression in the quadriceps muscle. These findings suggest that reduced irisin levels in chronic HF may serve as an indicator of disease progression [108].

### 3.8. Biomarker Relevance in HFpEF Versus HFrEF

Despite their different pathophysiological backgrounds, both HFpEF and HFrEF are characterized by significantly elevated levels of NT-proBNP, with a more pronounced increase in HFrEF, reflecting higher degrees of cardiac stress and volume overload. ST2, a prognostic biomarker predominantly studied in HFrEF, is markedly elevated in this subgroup and is associated with myocardial stress and fibrosis, suggesting an important role in assessing disease severity [14].

Inflammatory biomarkers also demonstrate phenotype-specific differences. hsCRP was reported to be significantly higher in HFrEF compared with HFpEF patients [109]. Furthermore, large-cohort analyses have identified biomarker patterns that distinguish the risk of incident HF phenotypes. NT-proBNP, GDF-15, ADM and CRP were associated with an increased risk of developing HFpEF. In contrast, MPO showed a stronger association with HFrEF, followed by cystatin-C, and NT-proBNP [110].

## 4. The Multimarker Approach in Heart Failure

The pathophysiological mechanisms involved in HF are often complex and intertwined, and using only one biomarker cannot reflect the full spectrum of hemodynamic stress, myocardial injury, fibrosis, inflammation, and cardiorenal dysfunction that determines disease prognosis. Therefore, a multimarker approach has been proposed to provide a more comprehensive assessment and improve risk stratification.

In a chronic HF cohort, van der Stam et al. demonstrated that NT-proBNP, sST2 and GDF-15 were independent predictors of the composite outcome (HF hospitalization, ICD shock or death). The authors combined these three biomarkers into a 0–3 point Heartmarker score (1 point for each biomarker above its cutoff), stating that patients with a score of 2–3 had significantly worse event-free survival than those with 0–1; moreover, the multimarker score outperformed the NYHA functional class in discriminating risk classes [111].

In an HFpEF cohort derived from the TOPCAT trial, Chirinos et al. measured 49 plasma biomarkers and identified several predictors of adverse outcomes, including FGF-23, FABP-4, IL-6, GDF-15, ST2, NT-proBNP, angiopoietin-2, and MMP-7. Using a machine-learning-based model, the authors demonstrated a strong association with the composite outcome of all-cause death or HF-related hospitalization (HR: 2.85; 95% CI: 2.03–4.02; *p* < 0.0001), while the multimarker approach improved risk prediction when added to the MAGGIC score, and outperformed it as an independent prognostic tool [43]. Analyzing the same TOPCAT trial dataset, Cohen et al. identified three distinct clinical phenogroups with different echocardiographic characteristics and biomarker profiles. Phenogroup 1 included younger patients with preserved functional status, low NT-proBNP, and higher MMP-9 and syndecan-4, showing minimal LV remodeling. Phenogroup 2, composed of predominantly female patients with atrial fibrillation and CKD, displayed a small left ventricle with concentric remodeling and the largest left atria, with increased biomarkers of innate immunity and vascular calcification, including osteoprotegerin, TIMP-4, IL-8, and ICAM-1. Phenogroup 3, characterized by obesity, diabetes, and CKD, had concentric ventricular hypertrophy, higher filling pressures, and elevated biomarkers of TNF-α-mediated inflammation (TNF-α, soluble TNF receptors type 1 and 2), intermediary metabolism (FABP-4, FGF-21, GDF-15), mineral metabolism (FGF-23, osteoprotegerin), liver fibrosis (YKL-40), renal injury (cystatin C), and tissue remodeling (sST2). Prognosis differed markedly, with phenogroup 3 showing the highest risk of cardiovascular death, HF hospitalization, or cardiac arrest (HR 3.44, 95% CI 2.79–4.24) compared with phenogroup 1, while also demonstrating the most pronounced benefit of spironolactone treatment [112].

Even combining biomarkers from the same pathophysiological pathway, such as inflammation, can significantly improve prognostic assessment. In a chronic HF cohort, Traxler et al. evaluated a panel of sST2, heat shock protein 27 (HSP27), and hsCRP, showing that patients with two or more elevated biomarkers had a substantially higher risk of cardiovascular death or unplanned HF hospitalization compared with those with none or one; the multimarker panel remained an independent predictor after adjustment for conventional risk factors and NT-proBNP [113].

In three independent HFpEF cohorts, Michaëlsson et al. identified 33 circulating biomarkers consistently associated with adverse outcomes. The study identified five strong predictors of HF hospitalization or all-cause death, including inflammatory biomarkers (TNFR-1, TNF-related apoptosis-inducing ligand (TRAIL)-receptor 2), markers of cardiac remodeling (GDF-15, uPAR), and ADM, a biomarker of neurohormonal activation. Additional markers, such as FABP-4, and FGF-23, were associated with impaired functional capacity and poor quality of life. Importantly, many of these prognostic biomarkers were down-regulated following treatment with an MPO inhibitor, highlighting inflammatory and remodeling pathways as both prognostic markers and therapeutic targets in HFpEF [114].

In stable chronic HF, Dupuy et al. investigated fibrosis-related biomarkers reflecting collagen synthesis (procollagen type I N-terminal propeptide [PINP], PIIINP), degradation (c-terminal telopeptide of collagen type I [CTx]), and regulatory mediators (sST2, galectin-3). Among them, the CTx/PIIINP ratio and sST2 were independent predictors of mortality, and their combined elevation conferred a more than fivefold increased risk of death [115]. By contrast, in the acute setting, Gao et al. analyzed 18 circulating biomarkers, demonstrating positive correlations with 2-year all-cause mortality for NT-proBNP, hs-TnT, GDF-15, TNF-α, endoglin, TIMP-1, MMP-2 and MMP-9; TIMP-1 exhibited the strongest association, highlighting the importance of extracellular matrix turnover in this cohort. A machine-learning model combining the full biomarker panel with clinical variables achieved high predictive accuracy (AUC 0.83 in training and 0.80 in validation), significantly outperforming conventional clinical models [116].

Just as no single biomarker can fully capture HF prognosis, multiple studies suggest that combining biomarkers can also improve the diagnosis of HF in both acute and chronic settings. In the acute HF setting, NPs remain central for identifying HF, but adjunct markers have shown additive value. For example, in older emergency patients with acute dyspnea, adding novel markers of vascular tone to NT-proBNP significantly improved the discrimination of acute HF. Bahrmann et al. found that combining MR-proADM with NT-proBNP modestly increased the C-index (from 0.81 to 0.84, *p* = 0.045) and improved reclassification metrics for acute HF diagnosis. Similarly, adding C-terminal pro-endothelin-1 (CT-proET-1) yielded an even higher C-index (0.86 vs. 0.81, *p* = 0.031) and robust net reclassification improvement. Overall, patients with both an elevated NPs and a second biomarker had a much higher probability of acute HF than those with only one elevated marker, underscoring that dual-marker approaches outperform single markers in the acute setting [117].

In chronic HF and ambulatory settings, a combined biomarker strategy may aid in diagnosing HF, especially HFpEF, where single markers are often less definitive. NPs are still the cornerstone for chronic HF diagnosis, but their levels can be modest in HFpEF or in patients with obesity, and additional biomarkers reflecting fibrosis or inflammation can strengthen diagnostic confidence. For instance, a recent study assessing patients undergoing catheterization demonstrated that pairing NT-proBNP with sST2 improved identification of elevated left ventricular filling pressures. Călburean et al. showed that NT-proBNP and sST2 were both independent predictors of high LV end-diastolic pressure, and the combination of the two significantly enhanced the detection of diastolic dysfunction in a multivariable model [118]. Moreover, emerging research using high-throughput proteomics has identified multi-biomarker panels that outperform NPs alone in recognizing incipient HF. In one machine-learning analysis, adding novel proteins to BNP greatly improved the classification of HF vs. non-HF patients. Notably, a panel of BNP plus IGF-2 and inter-α-trypsin inhibitor heavy chain H3 (ITIH3) achieved an AUC of ~0.93 for distinguishing HF with preserved EF from controls, compared to ~0.88 with BNP alone. Likewise, combining BNP with markers of inflammation markedly boosted the detection of HFrEF in that study [119].

Beyond the advances in circulating biomarkers, novel approaches are being explored at the interface between cardiology and materials science. In particular, conductive polymers such as poly(3,4-ethylenedioxythiophene):poly(styrenesulfonate) (PEDOT:PSS) have shown promising properties in preclinical models. These biomaterials exhibit excellent biocompatibility and electroconductive features that support cardiomyocyte coupling, reduce electrical heterogeneity, and have been reported to attenuate arrhythmic events in vitro. Recent studies demonstrated that PE-DOT:PSS-based platforms can modulate cardiac electrophysiology and potentially prevent the onset of malignant arrhythmias, thereby opening new translational directions for HF management [120,121]. Although still at an early experimental stage, such strategies illustrate the potential of integrating molecular biomarkers with cutting-edge biomaterials and bioelectronics to improve patient out-comes.

The routine biomarkers currently approved and widely used in HF for diagnosis, prognosis, and treatment monitoring are NPs (BNP and NT-proBNP) and hs-cTn. These remain the universal biomarkers for diagnosis, risk stratification, and prediction of cardiovascular death, all-cause mortality, and HF-related outcomes in patients across both HF phenotypes. In addition, markers of fibrosis and inflammation, such as sST2 and Gal-3, have demonstrated prognostic value and the ability to enhance the predictive capacity of NPs in HF patients, regardless of cardiovascular risk factor burden or HF phenotype [122]. However, despite their clinical relevance, the use of sST2 and Gal-3 in routine practice is less widespread than that of NPs and Tn.

Although multiple novel biomarkers are under investigation for their diagnostic and prognostic utility in HF, none have yet demonstrated sufficient specificity for the disease, and thus are not recommended for routine clinical use. Among these emerging biomarkers, GDF-15 appears to provide additional prognostic information regarding HF morbidity and mortality [68].

## 5. Conclusions

HF remains a major clinical challenge due to its rising prevalence and significant impact on mortality and quality of life. A deeper understanding of its complex pathophysiology, encompassing inflammation, oxidative stress, cardiac remodeling, and neurohormonal activation, and genetic and environmental factors, has enabled the identification and validation of a wide range of biomarkers with diagnostic and prognostic value. These biomarkers not only provide crucial insights into disease stage and progression but also offer opportunities for a more personalized approach to patient management. Their integration into routine clinical practice represents an important step toward optimizing therapeutic strategies and improving long-term outcomes; however, further studies are still required to validate and standardize their use across different patient subgroups.

Future research should also focus on validating multimarker strategies in large, well-phenotyped HFpEF and HFrEF cohorts, as well as on integrating circulating biomarkers with advanced imaging modalities to refine patient stratification. Moreover, the incorporation of artificial intelligence and machine learning tools into biomarker and imaging datasets may significantly enhance predictive accuracy and support individualized therapeutic approaches.

## Data Availability

No new data were created or analyzed in this study. Data sharing is not applicable to this article.
